# Home as a Place of Noise Control for the Elderly? A Cross-Sectional Study on Potential Mediating Effects and Associations between Road Traffic Noise Exposure, Access to a Quiet Side, Dwelling-Related Green and Noise Annoyance

**DOI:** 10.3390/ijerph15051036

**Published:** 2018-05-21

**Authors:** Natalie Riedel, Heike Köckler, Joachim Scheiner, Irene van Kamp, Raimund Erbel, Adrian Loerbroks, Thomas Claßen, Gabriele Bolte

**Affiliations:** 1 Department of Social Epidemiology, Institute of Public Health and Nursing Research, University of Bremen, Grazer Straße 4, 28359 Bremen, Germany; gabriele.bolte@uni-bremen.de; 2 Institute of Occupational, Social, and Environmental Medicine, Centre for Health and Society, Faculty of Medicine, University of Düsseldorf, Universitätsstraße 1, 40225 Düsseldorf, Germany; adrian.loerbroks@uni-duesseldorf.de; 3 Department of Community Health, Hochschule für Gesundheit (University of Applied Science), Gesundheitscampus 6–8, 44801 Bochum, Germany; heike.koeckler@hs-gesundheit.de; 4 Department of Transport Planning, Faculty of Spatial Planning, TU Dortmund University, August-Schmidt-Str. 10, 44221 Dortmund, Germany; joachim.scheiner@tu-dortmund.de; 5 Centre for Sustainability, Environment and Health, National Institute for Public Health and the Environment (RIVM), Antonie van Leeuwenhoeklaan 9, 3721 MA Bilthoven, The Netherlands; irene.van.kamp@rivm.nl; 6 Institute of Medical Informatics, Biometry and Epidemiology, Essen University Hospital, Hufelandstr. 55, 45147 Essen, Germany; raimund.erbel@uk-essen.de; 7 Centre for Health NRW (North Rhine-Westphalia), Section “Health Assessments and Forecasting”, Gesundheitscampus 10, 44801 Bochum, Germany; thomas.classen@lzg.nrw.de

**Keywords:** noise annoyance, perceived noise control, road traffic noise exposure, quiet side, dwelling-related green, mediation analysis, Heinz Nixdorf Recall Study

## Abstract

Urban residents’ need to be in control of their home environment can be constrained by perceived uncontrollability of exposure to road traffic noise. Noise annoyance may indicate a psychological stress reaction due to this uncontrollability perception, thereby undermining the restoration process. Environmental resources, such as having access to a quiet side at home and dwelling-related green, may reduce noise annoyance both directly by shielding acoustically and indirectly by enhancing residents’ perceived noise control. We assessed the potential mediating role of perceived noise control in independent and joint associations of road traffic noise exposure (>65 dB L_den_) and of an absent dwelling-related environmental resource (three indicators concerning quiet sides and one indicator concerning dwelling-related green) with noise annoyance. In our cross-sectional, questionnaire-based study on elderly urban citizens (*N* = 1812), we observed a statistically significant indirect effect of noise exposure on noise annoyance through perceived noise control (39%, 95%CI 26–55%). Statistical mediation between indicators of absent environmental resources and noise annoyance was weaker. The potential indirect effect was confirmed for combinations of noise exposure with each of the four indicators of an absent environmental resource. Our findings may call for mitigating noise levels while fostering quietness and green at residents’ homes.

## 1. Introduction

The home is meant to be a place where residents ideally should be in control of their immediate environment, to pursue any activity without constraints from external stressors and uncontrollable circumstances, to feel comfortable, safe, and at ease. The term “perceived control” in this article represents affective attributes, including a sense of control, autonomy, safety, constancy, privacy, retreat, or freedom. In the literature, these aspects of a home are referred to as ontological security [1] or as psychosocial benefits [2,3] and have been related to residential satisfaction [4]. Perceived control is shaped by perceived housing conditions like noise, dampness, temperature, space, or maintenance [2,3,4,5]. Hence, perceived control at home has been proposed as a mediator in the relationship between housing conditions and mental health [6]. Reduced control at home has been linked to, for example, self-rated poor health [7], unwell-being [4], and depression and anxiety [8].

Road traffic noise is an external stressor potentially affecting residents in their homes. Its prevalence in urban areas has been receiving increasing attention in environmental health research [9], as well as European environmental politics [10]. This is underpinned by recent systematic reviews on exposure-response relations in the context of the Environmental Noise Guidelines development process [11] and the impending revision of the European Environmental Noise Directive (END) [12]. The END introduces noise annoyance as a focal health indicator in relation to chronic noise exposure. Besides its overall public health relevance [9] and the apparent need to revise previous annoyance assessments [13,14], noise annoyance represents a pivotal outcome concerning residents’ perceived noise control at home, as discussed below.

Noise annoyance is closely related to the concept of road traffic noise as an “ambient stressor” [15]. Its psychophysiological implications have been causally attributed to exposure-specific uncontrollability and unpredictability in reference to the concept of learned helplessness [5,16,17,18]. These exposure characteristics are particularly pronounced in urban settings, where traffic flows are frequently disrupted (e.g., at signal-controlled intersections or due to traffic congestion on major roads). We have suggested a causal link from chronic noise exposure via perceived uncontrollability of personal noise exposure (hereafter: perceived noise control) to noise annoyance [19]. While classifying perceived noise control as a non-acoustic (psychosocial) determinant of noise annoyance [20], previous research has explained its impact on health and noise annoyance by the secondary appraisal construct (partly) in reference to Lazarus stress model [21,22,23]. As a consequence, the association of objective exposure to road traffic noise with perceived noise control has not been studied yet. Though unmeasured, perceived noise control was referred to as an explanatory mechanism in studies demonstrating adverse health effects, including somatic symptoms and self-rated health, due to traffic noise exposure and traffic noise-related annoyance [24,25,26]. In a similar vein, research dealing with the concept of restorative environments and soundscapes has recognised the meaning of home as a place of perceived control [27,28]. It argues in compliance with Attention Restoration Theory [29] that traffic noise exposure impedes residents’ renewing attentional resources by imposing a state of sustained arousal and vigilance [28,30]. Thus, resources required for concentration and self-directed activities are not replenished, which translates into compromised stress recovery, restricts behavioural options and leads to a growing restoration need. Arguments from both concepts (noise-induced helplessness and restoration) allowed us to assume that socially unfavourable behavioural patterns like irritability, resignation, withdrawal, and disinterest in the neighbourhood environment are more likely to occur [19] because cognitive capacities are impaired by prolonged psychophysiological adaption to the traffic-related noise stressor [24,28].

Meanwhile, both noise epidemiology and noise-related restoration research have suggested beneficial effects of environmental resources located at the microlevel of residents’ dwelling (home), that is, having access to a quiet building side and dwelling-related green (e.g., backyard) [31,32,33,34,35,36,37,38,39,40]. Besides potential direct effects on health, these resources may alleviate noise annoyance by mitigating noise exposure (acoustic shielding). Psychologically, they may be conducive to the restoration process by providing an audio-visually fascinating scenery and masking traffic noise with more pleasant sounds. While allowing refuge and respite from traffic noise exposure and enhancing perceived noise control at home, environmental resources may enable residents to develop a sense of control in accordance with the notion of home described above. Consequently, residents may have enough attentional resources to engage in activities selected to reach more long-term personal goals than just struggling to cope with the personal noise exposure in the short term [28]. In the presence of environmental resources, noise annoyance is therefore likely to decrease. Conversely, the absence of these environmental resources may induce fatigue and psychological distress that may add to residents’ noise annoyance or even enhance their vulnerability to chronic exposure to road traffic noise.

**Figure 1:**
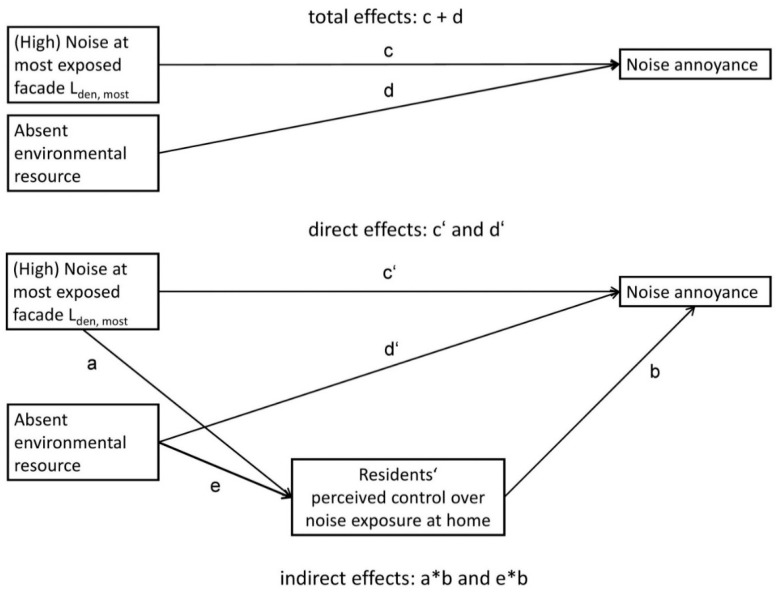
Associations under study for the first objective. Shown are potential total, direct, and indirect effects we aimed to quantify.

**Figure 2:**
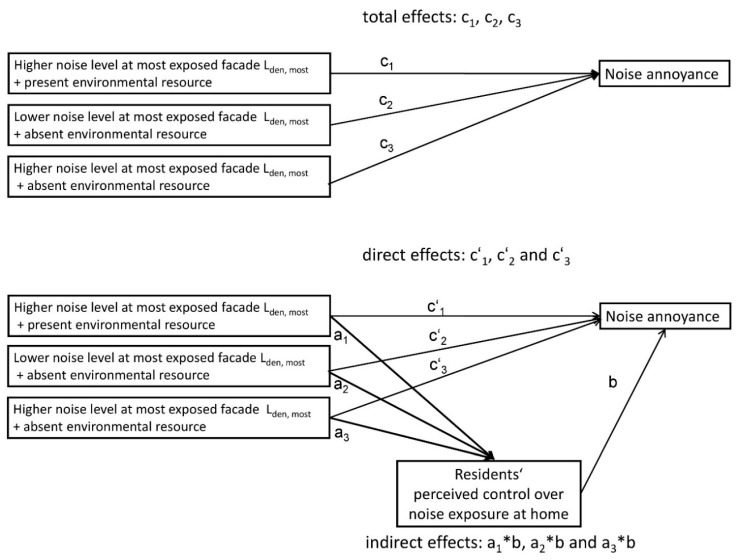
Associations under study for the second objective. Shown are potential total, direct, and indirect effects we aimed to quantify.

Against this background, we pursued two objectives:

1. To investigate whether the independent associations of exposure to road traffic noise and of absent dwelling-related environmental resources with noise annoyance are significantly mediated by perceived noise control (Figure 1).
2. To explore joint associations of road traffic noise exposure levels and present/absent dwelling-related environmental resources with perceived noise control, as well as noise annoyance (Figure 2), assuming that the double burden of higher noise levels and absent dwelling-related environmental resources is statistically associated with a particularly marked decrease in perceived noise control and increase in noise annoyance. We hypothesised that the potential mediating pathway through perceived noise control would be more pronounced under these double burden conditions.

We addressed these two research objectives using cross-sectional data from a population-based sample of elderly citizens living in an urban agglomeration.

## 2. Materials and Methods

### 2.1. Study Population of Our Cross-Sectional Study

In 2016, we conducted a survey among participants of the Heinz Nixdorf Recall (HNR) study, an ongoing epidemiological cohort initiated in 2000 in the three neighbouring cities of Mülheim, Essen, and Bochum. These German cities are located in the densely populated Ruhr area that is shaped by considerable economic restructuring, from coal and steel to new sectors, and by huge differences in environmental quality, as well as in social characteristics and health status of its population. Originally, the HNR study included about 4800 women and men who had been randomly selected from population registries and were aged 45–75 years at the time of baseline examination (2000–2003). The HNR study was ethically approved by the institutional review boards of the Ethical Commission of the Medical Faculty of the University of Duisburg-Essen, adheres to high quality standards as defined by DIN EN ISO 9001: 2000/2008, and is based on participants’ written consent [41]. Until now, study participants have undergone three comprehensive examinations (last wave: 2011–2014).

Using the annual follow-up dispatch, we were able to gather data from 2402 participants aged 60–90 years with a study-specific questionnaire in 2016. While achieving an overall high response rate of still active participants (ca. 83% of 2899), we had to exclude 24.6% (*N* = 590) for our complete case analysis. Main reasons for exclusion were that (1) participants had moved from the study region (*N* = 138), (2) noise or land use data (see section on exposure measures below) were not available at participants’ address (*N* = 119), and (3) the participants did not complete relevant questionnaire items (*N* = 333). Thus, our final study sample size was *N* = 1812.

Our questionnaire data were first merged with original data from the HNR study in order to retrieve information on sociodemographic characteristics, as well as residential dissatisfaction and sleep quality (see below). Second, these individual data were linked to environmental data (road traffic noise and land use) to derive exposure measures.

### 2.2. Measures

#### 2.2.1. Exposure to Noise Exposure at the Most Exposed Façade, L_den,most_

We made use of the average noise levels emitted from road traffic that had been modelled at the most exposed façade of participants’ dwelling, as obliged by the European Environmental Noise Directive (END) [42] (noise indicator L_den_, referring to all days, evenings, and nights during one year, second round modelling in 2012). For our main analyses, we dichotomised the continuous L_den,most_ values at noise levels exceeding 65 dB L_den,most_. A lower cutoff point (>55 dB L_den,most_, threshold exposure level for noise mapping) and the continuous noise measure (per 10 dB, L_den,most_) were used for sensitivity analyses.

#### 2.2.2. Absent Dwelling-Related Environmental Resources (Quiet Side and Dwelling-Related Green)

We defined the absence of a quiet side and dwelling-related green by four indicators:

(a) Both living and sleeping room faced a street, as opposed to at least one of these rooms lying next to a courtyard, garden, park, field, or another building (questionnaire-based). The living room referred to the room mostly used by the participants. The location of rooms has been previously used in other studies to reduce exposure misclassification and to study the modification of relatively quiet sides on noise effects [33,43].
(b) A signal-controlled intersection was present in front of the living and/or sleeping room (questionnaire-based). Such a layout of rooms may go together with more abrupt noise peaks in addition to usual average sound pressure levels, leading to a reinforcement of noise-related stress reactions [16]. We are aware that other right-of-way regulations may also cause abrupt noise. However, such regulations are typically used for minor roads with low traffic loads.
(c) The dwelling did not possess a façade where no more than 40 dB L_den_ prevailed (based on END noise modelling). This cutoff point for the least exposed façade L_den,least_ has been described as the threshold value for urban background noise [35] and appears as a baseline value for exposure-response functions [14]. Moreover, the combination of >65 dB L_den,most_ with ≤40 dB L_den,least_ corresponds to the definition of a “quiet façade” by the END (Annex IV), requiring a difference of at least 20 dB L_den_ between the most and least exposed dwelling façade.
(d) There was no dwelling-related green, as mapped by the Ruhr Regional Association in 2015. These land use maps have been successfully utilised for analyses on distributional environmental justice dealing with public green space (e.g., [44,45]). Given our focus on perceived noise control at home, we were interested in the land use categories containing dwelling-related green only.

#### 2.2.3. Composite Variables: Dichotomised Road Traffic Noise Exposure L_den,most_ Combined with Present/Absent Dwelling-Related Environmental Resources

Next, we combined the binary road traffic noise variable with each binary dwelling-related environmental variable ((a), (b), (c), (d)) separately, composing four categorical variables in order to analyse four exposure constellations, respectively:

- noise levels ≤65 dB L_den_ at the most exposed façade plus presence of dwelling-related environmental resource (reference category),
- noise levels >65 dB L_den_ at the most exposed façade plus presence of dwelling-related environmental resource,
- noise levels ≤65 dB L_den_ at the most exposed façade plus absence of dwelling-related environmental resource, and
- noise levels >65 dB L_den_ at the most exposed façade plus absence of dwelling-related environmental resource.

#### 2.2.4. Potential Mediator (Perceived Noise Control) and Outcome (Noise Annoyance)

Participants’ perceived noise control at home was captured by one single item: feeling helpless in relation to indoor noise exposure at home. Participants were asked to rate their agreement on a 6-point Likert scale, with higher values representing less perceived noise control (1 = do not agree at all, 6 = fully agree). A similar item was used in previous studies testing the relationship between perceived noise control and noise annoyance [23] or subjective health [46]. Annoyance was measured by one source-specific item and related to road traffic noise heard by participants “when their windows were closed” according to the “Large Analysis and Review of European Housing and Health Status” [47,48]. Using a 5-point Likert scale, as recommended by expert committees [14,49], participants indicated the degree of their annoyance (1 = not at all annoyed, 5 = extremely annoyed).

#### 2.2.5. Additional Predictors

Generally, sociodemographic characteristics have been rated as weak predictors of noise annoyance [50]. In the present analyses, we regarded gender, age at time of survey (in 2016), and education (categorised in ≤10, 11–13, 14–17, and ≥18 years of formal school and vocational training [51]).

In order to substantiate our statistical analyses, we considered additional predictors of perceived noise control and noise annoyance in accordance with our recently published theoretical model on cognitive-motivational determinants of noise-related health inequities against the background of European Noise policy (END) [19]. Furthermore, our selection of predictors was guided by previous research on housing and health (see Introduction above, as well as [47,48]), on procedural environmental justice [52], and on noise annoyance (e.g., [53,54]). This included the following variables:

- home ownership as opposed to living as a tenant, measured by a binary variable;
- residential dissatisfaction related to the neighbourhood, measured by one single item with a 4-point Likert scale indicating the degree of residential satisfaction and categorised into 1 = (very) dissatisfied and 0 = (very) satisfied. This item stemmed from the last HNR examination (see Section 2.1);
- participants’ wish to change their residence (yes vs. no), measured by a binary variable;
- noise sensitivity, measured by a sum score derived from nine items capturing participants’ agreement to reactions to sounds in different settings, as authored by [55]. Single items had a range from 0 to 3, yielding a potential sum score from 0 to 27 with a standardised Cronbach’s alpha = 0.60;
- learned (generalised) helplessness, measured by the mean score of two items with a 6-point Likert scale. Theoretically, we drew on the interpretation of helplessness as participants’ expectancy of general non-contingency between behaviour and outcome within the frame of the cognitive activation theory of stress (CATS) [56]. We constructed German items representing this type of helplessness, as inspired by the theoretically originated measure of the cognitive activation theory of stress (e.g., “I really don’t have any control over the most important issues in my life”) [57]. The standardised Cronbach’s alpha for our two items was 0.56;
- sleep quality, measured by the sum score of items from the Pittsburgh Sleep Quality Index [58]. Our sum score covered self-rated sleep quality (one single item), latency (one single item), duration (one single item), disturbance (subscore based on the sum of eight items describing the frequency of different reasons for disturbances, with Cronbach’s alpha = 0.63), sleeping medication (one single item), and daytime dysfunction (subscore based on the sum of two items, with Cronbach’s alpha = 0.37). Each of these components had a final range from 0 to 3, leading to a potential range from 0 to 18. The standardised Cronbach’s alpha for the sum of aforementioned components was 0.66. Sleep quality items were collected during the last HNR examination (see Section 2.1).

Higher values on the instruments for noise sensitivity, learned helplessness, and sleep quality indicated a greater affectedness, respectively.

#### 2.2.6. Covariates for Sensitivity Analysis on Exposure Differences

To assess exposure misclassification, we controlled for length of residency, window-opening habits, and floor level in further sensitivity analyses. A variable capturing length of residency was constructed based on the difference between the year of the data collection (i.e., 2016) and the year the participants reported to have moved to their current address. Given the skewness towards long residence duration (median: 31 years), we defined four categories: 0–5 years, 6–10 years, 11–31 years, >31 years. Window-opening habits referred to “most of the time” during summer and to either living or sleeping room (windows kept open vs. windows closed). As the noise indicator L_den_ is assessed at about 4 m aboveground according to the END (Annex 1) and sound pressure can be amplified by building facades, we built a binary variable informing about whether participants’ living or sleeping room was located on the first floor or higher.

### 2.3. Statistical Analyses

At first, we examined whether there were linear relations between the noise exposure variable L_den,most_, perceived noise control and noise annoyance. To this end, we calculated mean values of L_den,most_ and noise annoyance within the response categories of the perceived noise control item and, correspondingly, mean values of L_den,most_ and perceived noise control within the response categories of the noise annoyance item. Observing gradual increases of mean values within response categories in both instances, we continued to apply linear regression analyses as implemented in the macro-tool “Process”, version 2.16, by Hayes [59], that is programmed to assess indirect effects of intermediate variable(s) in the association between an exposure and outcome variable (mediation analysis). This tool is programmed to assess indirect effects rather conveniently using the bootstrapping technique. We decided to rely on 10,000 bootstraps to estimate 95% confidence intervals for the indirect effect of noise exposure levels and environmental resources on noise annoyance through perceived noise control. Further, “Process” allowed us to determine indirect effects for our four-categorical composite variable (L_den,most_ + presence/absence of one of the environmental resources) [60]. In view of our cross-sectional study design, we do not consider statistical significance as a proof of causality, while adhering to the terminology of total, direct, and indirect effects as estimated by “Process”.

After studying crude relations (Model I) for the first research objective, participants’ sociodemographic characteristics were added to the model (Model II). Next, the regression model was extended by additional predictors (Model III) and, finally, by one of the four dwelling-related variables indicative of an absent environmental resource in subsequent models (Model IV). The same modelling strategy was applied to the sensitivity analysis on exposure misclassification, where we additionally adjusted for the covariates length of residency, window-opening habits, and floor level across Models I–III. Analyses for the second objective, using the composite variable, was based on the extended Model III.

## 3. Results

### 3.1. Descriptive Statistics (Table 1 and Table 2)

Both genders were equally distributed and, on average, participants were 71 years old (Table 1). About 43% lived as a tenant. Most participants were satisfied with their neighbourhood, while more than 7% would like to change their residence.

Mean scores of noise annoyance (1.39) and perceived noise control (1.49) indicated a low prevalence of noise annoyance and a high prevalence of perceived noise control (Table 2). About 13% of the participants were exposed to high noise levels at the most exposed façade of their dwelling (>65 dB L_den,most_), whereas exposure prevalence amounted to 42% (*N* = 768) at the lower cutoff point (>55 dB L_den,most_, not shown in Table 2). In bivariate statistics, both outcome and mediator variable were significantly elevated in the higher noise exposure strata (Table 2). Moreover, participants at higher exposure levels were more likely to miss a quiet side, as indicated by rooms facing an intersection, increased background noise levels at the least façade (>40 dB L_den,least_), and lacking green at their dwelling.

### 3.2. Results for the First Research Objective: Independent Associations and Statistical Mediation (Figure 1, Table 3, Table 4, Table 5, Table 6 and Table 7)

Figure 1 illustrates the associations that were estimated for our first research objective. Table 3, Table 4, Table 5, Table 6 and Table 7 are structured correspondingly. The potential indirect effects of L_den,most_ and absent environmental resources on noise annoyance transmitted by perceived noise control are given at the bottom of the tables.

In Model I, noise exposure levels exceeding 65 dB L_den,most_ were related to an increase in noise annoyance in the total effect model (Table 3: c = 0.45, 95%CI (0.35–0.55)). A more distinct effect was estimated for the association between L_den,most_ at this cutoff point and perceived noise control (a = 0.56, 95%CI 0.42–0.70). Including perceived noise control in the model on noise annoyance (b = 0.36, 95%CI (0.33–0.39), the association of L_den,most_ with noise annoyance was reduced, but remained significant (c’ = 0.25, 95%CI (0.16–0.33)). This implies that perceived noise control explains almost 45% of the association of L_den,most_ with noise annoyance. Adjustment for sociodemographic characteristics merely altered effect estimates for noise annoyance in Model II (results not shown in Table 3). In Model III, effect estimates were slightly attenuated by additional predictors, which resulted in a point estimate for the indirect effect of 39%.

Accounting for the absence of an environmental resource (Model IV based on Model III in Table 4, Table 5, Table 6 and Table 7) did not change our findings. Each of the four variables indicative of the lacking resource was independently associated with both perceived noise control and noise annoyance (see coefficients d, d’, and e). Statistical mediation occurred to a lesser extent, with indirect effects ranging from 26% (Table 6, indicator >40 dB L_den,least_) to 37% (Table 5, indicator intersection) on average.

The partial mediation through perceived noise control was statistically confirmed in sensitivity analyses using

- the lower noise exposure cutoff point of >55 dB L_den,most_ (37% in Model I, 32% in Model III),
- the continuous noise exposure L_den,most_ per 10 dB (range: 33–79 dB; 42% in Model I, 37% in Model III), and
- length of residency, window-opening habits, and floor level as additional covariates (46% in Model I, 40% in Model III) (results for coefficients available upon request).

### 3.3. Results for the Second Research Objective: Joint Associations and Statistical Mediation (Figure 2, Table 8, Table 9, Table 10 and Table 11)

In line with Figure 2, Table 8, Table 9, Table 10 and Table 11 display the results for our statistical mediation analysis based on our composite variables combining noise levels and (absent) environmental resources (as measured by the four dwelling-related indicators (a)–(d)). Accordingly, Table 8, Table 9, Table 10 and Table 11 show three effect coefficients estimated in relative comparison to the reference category (noise levels ≤65 dB L_den_ at the most exposed façade plus presence of the respective dwelling-related resource) for each association shown in Figure 2.

Except for the exposure constellation with the intersection indicator (Table 9), noise exposure levels >65 dB L_den,most_ (effect coefficients c_1_, ranging from 0.29 in Table 10 to 0.38 in Table 8) were more strongly related to noise annoyance than the lacking of environmental resources (effect coefficients c_2_, ranging from 0.14 estimated for the absent quiet side >40 dB L_den,least_ in Table 10 to 0.21 estimated for rooms facing streets in Table 8 in the total effect models). Despite its low prevalence, the single exposure condition “intersection in front of the living and/or sleeping room” was linked to a relatively large effect (c_2_ = 0.51), as compared to the traffic noise exposure >65 dB L_den,most_ (c_1_ = 0.31). Across all total effect models, double burdens produced the most prominent associations with noise annoyance, suggesting (at least) additive effects (effect coefficients c_3_). This pattern remained stable after including perceived noise control (direct effect model), although the strength of associations was diminished. Overall, results for the models on the association of L_den,most_ with perceived noise control (effect coefficients a_1_–a_3_) followed the same pattern, with double burdens yielding the most pronounced effects.

Double burdens did not necessarily translate into a particularly marked contribution of perceived noise control to the association of L_den,most_ with noise annoyance (Table 8, Table 9 and Table 10). This assumption held true only in the additional absence of green related to participants’ dwelling (Table 11), although this indirect effect size was just comparable to those indirect effects we observed for our first research objective above. At the same time, the indirect effect of lacking green through perceived noise control failed to reach statistical significance at the 95% level (28%, Table 11).

## 4. Discussion

In the present study, we set out to assess the potential mediating role residents’ perceived noise control is assumed to play in the association between road traffic noise exposure, (absent) dwelling-related environmental resources, and noise annoyance. Results from our cross-sectional data corroborate that perceived noise control could be a relevant psychological mechanism in the associations under study. While perceived noise control has been conceived as a psychosocial determinant of noise annoyance [20], its contribution to the restoration process has been suggested, but hardly empirically studied [31]. Thus, an increasing body of research shows how environmental resources, like having access to a quiet side and green, attenuate noise-related stress reactions without validating perceived noise control as an explanation [37,38,40]. Only recently, there is growing interest in exploring intermediate pathways, promoted by both greenspace and noise/soundscape researchers [31], and advancing a more comprehensive research agenda going beyond single exposure-response functions [61,62,63], as applied by current END policy and practice [64]. In this respect, our study added further evidence on how the concurrence of environmental stressors and unavailable resources can have cumulative impacts on both perceived noise control and noise annoyance. This underlines the need to contextualise potential cascades of psychological constraints and stress reactions, as illustrated by the notion of “loss cycles” related to environmental health [19,25,52], in reference to Hobfoll’s theory on the Conservation of Resources [65]. Moreover, if the location of relevant rooms at a signal-controlled intersection is an appropriate proxy for unpredictable noise events, the strong effect estimates we observed may call for a revision of noise exposure assessments that tend to solely rely on average sound pressures. Accordingly, there is an ongoing scientific debate on several noise indicators and their explanatory power for health effects [66].

In this article, we emphasised the meaning of home as an intended place of control that we conceptually linked to external ambient stressors (road traffic noise exposure) and dwelling-related environmental resources (having access to a quiet side and dwelling-related green). However, the ontological notion of home also encompasses psychosocial benefits clustering around status and self-identity [1,2,3] that we have not considered in our study. Exposure to road traffic noise might undermine residents’ sense of status and self-identity through perceived degradation of the residential address, which could additionally explain noise annoyance, as well as entail loss of self-esteem. Correspondingly, high noise exposure levels have been identified as a devaluing factor in housing market studies [61,67].

At the same time, environmental resources can confer higher living standards. Research on restorative soundscapes, quiet sides, and green space have pointed to “attractive quiet courtyards” [35] as an ingredient for positive affect and self-regulation, and social interaction [31,38,68]. Our measures of (not) having access to a relatively quiet side and dwelling-related green do not contain any information on objective (e.g., vegetation density relevant for diffraction of environmental noise) or subjective quality (e.g., perceived usefulness), which might have resulted in an underestimation of associations. In this line, scientists have started to qualify “restorativeness” using residents’ or interviewers’ ratings [30,69,70]. However, our study gives credit to simple indicators that are easy for urban planners to retrieve from maps on existing land uses (dwelling-related green and transport planning (signal-controlled intersection)), whereby noise interventions could be facilitated.

A further limitation relates to the age range and noise annoyance prevalence in our sample. Noise annoyance has been shown to be higher in middle-aged samples [71]. By way of comparison, a representative survey recounted 48% of the population aged 14+ to be at least moderately annoyed by road traffic noise in Germany, though without restricting the noise annoyance to the conditions “at home” and “when windows are closed” [72]. Despite low noise annoyance in our sample, the prevalence of noise levels exceeding 55 dB L_den_ was even higher than the average proportion of exposed residents reported for the second round of noise modelling in the three cities of our study region [10]. It is hypothesised that receding sensory acuity and decreasing stress arising from other life domains (work and family obligations) may account for less noise annoyance from late middle age onwards [71]. Environmental stress responses and stressors related to personal projects or the job may unfold additive [26] or even interactive [24] effects on health. In older age, morbidity may pose a much greater obstacle to everyday life than perceived health risks due to road traffic noise, given a high prevalence of chronic conditions in our sample (75% suffered from at least one chronic condition, i.e., diseases of joints, spines or muscles, asthma, diabetes, cancer, coronary heart disease, or stroke). Still, we found statistically significant associations with perceived noise control and noise annoyance, pointing to future research needs: for example, in-depth exploration of the interplay between physical constraints, perceived confinement to the dwelling, noise-related controllability at home, and subsequent stress reactions, as well as the replication of associations in younger age groups in order to model social vulnerabilities across different stages in the life course.

Another explanation for low noise annoyance levels in our study could be linked to long residence durations (median years spent at the current address: 31), a comparably high proportion of home owners (nearly 57% in our sample in 2016 vs. 46% on average in Germany and 43% in the federal state North Rhine-Westphalia of our study region in 2014 [73]) and an extremely high percentage of participants satisfied with their residential environment (>95%). These sample characteristics might suggest an overall contentment with life choices, including the residential location, as well as an inclination to habituate to noise readily [15]. In our sensitivity analysis on exposure differences, there was no trend towards higher annoyance among participants who had lived long at the residential address (as analysed using the categories described in the Method section and controlling for noise sensitivity, results for covariates not shown). By contrast, living as a tenant was associated with a somewhat higher noise annoyance (estimated coefficient 0.08, *p* < 0.05 in the total effect model; 0.06, *p* < 0.1 in the direct effect model), whereas there was no association with perceived noise control. Home ownership was shown to contribute to the sense of status [1,2]. However, its contribution was largely explained by “feeling happy about the home in general, living in an area with nice neighbours and a good reputation, fewer problems with the home (…) and owning more consumer durables” [2] (p. 405). Correspondingly, residential dissatisfaction and the wish to change the residence emerged as more relevant predictors of both perceived noise control (estimated coefficients 0.63, *p* < 0.0000 and 0.59, *p* < 0.0000, respectively) and noise annoyance (estimated coefficients 0.20, *p* > 0.05 and 0.16, *p* > 0.05, respectively, in the indirect effect model) than home ownership, which calls for research on underlying pathways, such as the impact of landlord relations on reduced perceived noise control among tenants [3].

We used noise data from just one point in time (as modelled by 2012) and linked it with individual data collected 4 years later. Changes in exposure levels due to noise abatement measures could have occurred since, which might have been accounted for in the END third round noise modelling officially finalised in 2017. Reviews on intervention effects have highlighted an excess response to both increases and decreases in changes in (road) traffic noise exposure levels, that is, observed changes in annoyance exceeded change values as expected from exposure-response functions under steady-state conditions [32,74]. It was beyond the scope of this study to track changes in noise exposure levels at residential addresses, though.

Cross-sectional study designs have been discussed as inadequate to model the causal sequence hypothesised in mediation analyses, unless reasoning and measurement of exposure, mediator, and outcome variables already imply a temporal order [75]. In this line, we may argue that perceived noise control and noise annoyance referred to participants’ current dwelling in our questionnaire, making it unlikely that these two psychological responses preceded the exposure to road traffic noise reported earlier for the respective residential addresses (see above). Further, we may expect noise annoyance to succeed perceived noise control closely in intraindividual processing, probably requiring a narrow time interval of measurements. Aware of this study limitation, we do not intend to regard our findings as proof of cause and effect, but rather as an exploratory approach to potential causal relations that future studies may build on. From this perspective, our mediation analysis appears as legitimate [76].

It is a strength of our study that we were able to consider a set of additional predictors based on a theoretical model [19]. Perceived noise control is placed at its core and linked to psychophysiological vulnerability to traffic-related noise on the one hand, and to civic engagement as envisaged by END noise action planning (i.e., intervention planning) on the other hand. A key assumption in this model is the generalisation of helplessness learned from (noise) uncontrollability experiences in different contexts (and vice versa: estimated effect of generalised helplessness on perceived noise control in this study: 0.13, *p* > 0.0000), affecting residents’ responsiveness to environmental stressors and readiness for proactive behaviour. Sustained arousal due to chronic noise exposure is likely to preclude residents from directing their attention to more long-term endeavours like civic engagement. Referring to restorative soundscapes, environmental planning may progress by creating urban spaces that help residents regain control over their states of mind [28] and spend cognitive capacities on engaging with their environment and planning processes. Regarding the overall robustness of associations in this study, our additional predictors seem to be rather part of causal relations, instead of acting as mere confounders, demanding a more complex analytical approach to study potential causal interrelations in the next step.

## 5. Conclusions

If confirmed elsewhere and in longitudinal studies, our findings may stress the need for planners to reduce noise levels in densely populated areas, as well as to maintain and foster quietness and dwelling-related green at residents’ homes. Noise annoyance palpably gives evidence of experiencing a lack of control that could interfere with the notion of home, as well as the END expectations of civic engagement. Thus, while trying to prove a psychological mechanism in a very first cross-sectional step, we practically assessed residents’ perceived control at home as an indicator of healthy housing conditions and civic engagement.

## Figures and Tables

**Table 1:** Characteristics of the study sample, *N* = 1812 ^1^.

Characteristics	Mean (SD)
Age, range 60–90	71.32 (6.95)
Learned helplessness, range 1–6	2.62 (1.36)
Noise sensitivity, range 2–27	14.80 (4.15)
Sleep quality (PSQI), range 0–16	4.82 (2.56)
	***N*** **(%)**
Female	894 (49.34)
Education	
≤10 years	112 (6.18)
10> years ≤13	989 (54.58)
13> years ≤18	455 (25.11)
>18 years	256 (14.13)
Home ownership: renting an apartment or house	782 (43.16)
Residential dissatisfaction (related to the neighbourhood)	78 (4.30)
Wish to change residence	138 (7.62)
Living or sleeping room on the first floor or higher (*N* = 1796)	1254 (69.82)
Window in one of the rooms open most of the time (*N* = 1803)	1658 (91.96)
Length of residency (*N* = 1769)	
>0–5 years	168 (9.50)
>5–10 years	136 (7.69)
>10–31 years	598 (33.80)
>31 years (median length)	867 (49.01)

^1^ If not indicated otherwise.

**Table 2:** Noise annoyance, perceived control and lack of dwelling-related environmental resources stratified by noise exposure *N* = 1812 ^1^.

Variable	All *N* = 1812		≤65 dB *N* = 1565 87.15%		>65 dB *N* = 247 12.85%		*p*-Value ^2^
	Mean	SD	Mean	SD	Mean	SD	
Noise annoyance, range 1–5	1.39	0.74	1.33	0.67	1.78	1.01	<0.0001
Perceived noise control, range 1–6	1.49	1.06	1.42	0.98	1.98	1.36	<0.0001
	***N***	**%**	***N***	**%**	***N***	**%**	
Both living and sleeping room faced a street.	621	34.27	526	33.61	95	38.46	0.1355
Intersection was in front of the living and/or sleeping room.	130	7.17	69	4.41	61	24.70	<0.0001
Noise level at the least exposed façade was >40 dB.	438	24.17	322	20.58	116	46.96	<0.0001
There was no dwelling-related green.	393 (*N* = 1786)	22.00	318	20.57	75	31.15	0.0002

^1^ If not indicated otherwise. ^2^ Based on χ^2^-test and Wilcoxon-Mann-Whitney test.

**Table 3:** Association of exposure to road traffic noise (>65 dB L_den,most_) with perceived noise control and noise annoyance ^1^.

*N* = 1812	Model I (Crude)				Model III (Fully Adjusted)			
	**Total effect of >65 dB L_den,most_ on noise annoyance**							
		Coeff.	95%CI lower	95%CI higher		Coeff.	95%CI lower	95%CI higher
Constant		1.3297	1.2936	1.3658		0.7837	0.4295	1.1379
>65 dB L_den,most_	c	0.4517	0.3539	0.5495	c	0.4014	0.3071	0.4957
	**Effect of >65 dB L_den,most_ on perceived noise control**							
		Coeff.	95%CI lower	95%CI higher		Coeff.	95%CI lower	95%CI higher
Constant		1.4173	1.3657	1.4688		1.0540	0.5567	1.5513
>65 dB L_den,most_	a	0.5625	0.4229	0.7021	a	0.4792	0.3468	0.6117
	**Direct effect of >65 dB L_den,most_ on noise annoyance**							
		Coeff.	95%CI lower	95%CI higher		Coeff.	95%CI lower	95%CI higher
Constant		0.8196	0.7695	0.8696		0.4397	0.1233	0.7561
>65 dB L_den,most_	c’	0.2492	0.1638	0.3345	c’	0.2450	0.1600	0.3300
Perceived noise control	b	0.3600	0.3323	0.3877	b	0.3264	0.2971	0.3557
	**Indirect effect of >65 dB L_den,most_ on noise annoyance through perceived noise control**							
		Coeff.	95%CI lower	95%CI higher		Coeff.	95%CI lower	95%CI higher
	a × b	0.2025	0.1357	0.2820	a × b	0.1564	0.1032	0.2223
	% ^2^	45%	30%	62%	%	39%	26%	55%

^1^ Shown are total, direct and indirect effects in the Model I (crude) and Model III (including home ownership, residential dissatisfaction, wish to change residence, noise sensitivity, learned helplessness, and sleep quality). ^2^ Percentage given by the formula (a × b/c) × 100.

**Table 4:** Association of exposure to road traffic noise (>65 dB L_den,most_) and absent quiet side (rooms located at street side) with perceived noise control and noise annoyance ^1^.

***N*** **= 1812**	**Total Effect of >65 dB L_den,most_ and of Rooms Located at Street Side on Noise Annoyance**			
		Coeff.	95%CI lower	95%CI higher
Constant		0.7696	0.4191	1.1200
>65 dB L_den,most_	c	0.3951	0.3017	0.4884
Both living and sleeping room facing a street	d	0.2168	0.1495	0.2841
	**Effect of >65 dB L_den,most_ and of rooms located at street side on perceived noise control**			
		Coeff.	95%CI lower	95%CI higher
Constant		1.0405	0.5456	1.5355
>65 dB L_den,most_	a	0.4732	0.3413	0.6050
Both living and sleeping room facing a street	e	0.2065	0.1114	0.3015
	**Direct effect of >65 dB L_den,most_ and of rooms located at street side on noise annoyance**			
		Coeff.	95%CI lower	95%CI higher
Constant		0.4375	0.1232	0.7519
>65 dB L_den,most_	c’	0.2441	0.1596	0.3286
Both living and sleeping room facing a street	d’	0.1509	0.0905	0.2113
Perceived noise control	b	0.3191	0.2899	0.3483
	**Indirect effect of >65 dB L_den,most_ on noise annoyance through perceived noise control**			
		Coeff.	95%CI lower	95%CI higher
	a × b	0.1510	0.0968	0.2174
	% ^2^	38%	25%	55%
	**Indirect effect of rooms located at street side on noise annoyance through perceived noise control**			
		Coeff.	95%CI lower	95%CI higher
	e × b	0.0659	0.0328	0.1028
	% ^3^	30%	15%	47%

^1^ Model IV based on Model III. ^2^ Percentage given by the formula (a × b/c) × 100. ^3^ Percentage given by the formula (e × b/d) × 100.

**Table 5:** Association of exposure to road traffic noise (>65 dB L_den,most_) and absent quiet side (intersection in front of sleeping or living room) with perceived noise control and noise annoyance ^1^.

***N*** **= 1812**	**Total Effect of >65 dB L_den,most_ and of Intersection in Front of Sleeping or Living Room on Noise Annoyance**			
		Coeff.	95%CI lower	95%CI higher
Constant		0.7769	0.4281	1.1258
>65 dB L_den,most_	c	0.3072	0.2111	0.4033
Intersection in front of the living and/or sleeping room.	d	0.4915	0.3635	0.6195
	**Effect of >65 dB L_den,most_ and of intersection in front of sleeping or living room on perceived noise control**			
		Coeff.	95%CI lower	95%CI higher
Constant		1.0461	0.5540	1.5382
>65 dB L_den,most_	a	0.3685	0.2330	0.5040
Intersection in front of the living and/or sleeping room.	e	0.5777	0.3971	0.7583
	**Direct effect of >65 dB L_den,most_ and of intersection in front of sleeping or living room on noise annoyance**			
		Coeff.	95%CI lower	95CI higher
Constant		0.4475	0.1334	0.7616
>65 dB L_den,most_	c’	0.1912	0.1044	0.2779
Intersection in front of the living and/or sleeping room	d’	0.3096	0.1936	0.4256
Perceived noise control	b	0.3149	0.2855	0.3443
	**Indirect effect of >65 dB L_den,most_ on noise annoyance through perceived noise control**			
		Coeff.	95%CI lower	95%CI higher
	a × b	0.1160	0.0643	0.1792
	% ^2^	38%	21%	58%
	**Indirect effect of intersection in front of sleeping or living room on noise annoyance through perceived noise control**			
		Coeff.	95%CI lower	95%CI higher
	e × b	0.1819	0.1002	0.2766
	% ^3^	37%	20%	56%

^1^ Model IV based on Model III. ^2^ Percentage given by the formula (a × b/c) × 100. ^3^ Percentage given by the formula (e × b/d) × 100.

**Table 6:** Association of exposure to road traffic noise (>65 dB L_den,most_) and absent quiet side (>40 dB L_den,least_) with perceived noise control and noise annoyance ^1^.

***N*** **= 1812**	**Total Effect of >65 dB L_den,most_ and of >40 dB L_den,least_ on Noise Annoyance**			
		Coeff.	95%CI lower	95%CI higher
Constant		0.7405	0.3876	1.0934
>65 dB L_den,most_	c	0.3577	0.2618	0.4536
>40 dB L_den,least_	d	0.1712	0.0947	0.2477
	**Effect of >65 dB L_den,most_ and of >40 dB L_den,least_ on perceived noise control**			
		Coeff.	95%CI lower	95%CI higher
Constant		1.0191	0.5218	1.5164
>65 dB L_den,most_	a	0.4439	0.3088	0.5790
>40 dB L_den,least_	e	0.1383	0.0305	0.2461
	**Direct effect of >65 dB L_den,most_ and of >40 dB L_den,least_ on noise annoyance**			
		Coeff.	95%CI lower	95%CI higher
Constant		0.4111	0.0954	0.7268
>65 dB L_den,most_	c’	0.2142	0.1279	0.3006
>40 dB L_den,least_	d’	0.1265	0.0582	0.1947
Perceived noise control	b	0.3232	0.2940	0.3524
	**Indirect effect of >65 dB L_den,most_ on noise annoyance through perceived noise control**			
		Coeff.	95%CI lower	95%CI higher
	a × b	0.1435	0.0908	0.2068
	% ^2^	40%	25%	58%
	**Indirect effect of >40 dB L_den,least_ on noise annoyance through perceived noise control**			
		Coeff.	95%CI lower	95%CI higher
	e × b	0.0447	0.0082	0.0884
	% ^3^	26%	5%	52%

^1^ Model IV based on Model III. ^2^ Percentage given by the formula (a × b/c) × 100. ^3^ Percentage given by the formula (e × b/d) × 100.

**Table 7:** Association of exposure to road traffic noise (>65 dB L_den,most_) and absent dwelling-related green with perceived noise control and noise annoyance ^1^.

***N*** **= 1786**	**Total Effect of >65 dB L_den,most_ and of Absent Green on Noise Annoyance**			
		Coeff.	95%CI lower	95%CI higher
Constant		0.7045	0.3475	1.0615
>65 dB L_den,most_	c	0.3903	0.2948	0.4859
Absent green	d	0.1673	0.0890	0.2455
	**Effect of >65 dB L_den,most_ and of absent green on perceived noise control**			
		Coeff.	95%CI lower	95%CI higher
Constant		0.9540	0.4514	1.4566
>65 dB L_den,most_	a	0.4736	0.3391	0.6081
Absent green	e	0.1806	0.0704	0.2908
	**Direct effect of >65 dB L_den,most_ and of absent green on noise annoyance**			
		Coeff.	95%CI lower	95%CI higher
Constant		0.3973	0.0777	0.7168
>65 dB L_den,most_	c’	0.2378	0.1515	0.3242
Absent green	d’	0.1091	0.0391	0.1791
Perceived noise control	b	0.3221	0.2925	0.3516
	**Indirect effect of >65 dB L_den,most_ on noise annoyance through perceived control**			
		Coeff.	95%CI lower	95%CI higher
	a × b	0.1525	0.0995	0.2184
	% ^2^	39%	25%	56%
	**Indirect effect of absent green on noise annoyance through perceived control**			
		Coeff.	95%CI lower	95%CI higher
	e × b	0.0582	0.0204	0.1003
	% ^3^	35%	12%	60%

^1^ Model IV based on Model III. ^2^ Percentage given by the formula (a × b/c) × 100. ^3^ Percentage given by the formula (e × b/d) × 100.

**Table 8:** Joint associations of exposure to road traffic noise (>65 dB L_den,most_) and present/absent quiet side (rooms located at street side) with perceived noise control and noise annoyance ^1^.

***N*** **= 1812**	**Total Effects of >65 dB L_den,most_ Combined with a Present/Absent Quiet Side on Noise Annoyance**			
		Coeff.	95%CI lower	95%CI higher
Constant		0.7731	0.4218	1.1245
≤65 dB L_den,most_ + quiet side (ref.)		0		
>65 dB L_den,most_ + quiet side	c_1_	0.3844	0.2663	0.5025
≤65 dB L_den,most_ + no quiet side	c_2_	0.2127	0.1400	0.2854
>65 dB L_den,most_ + no quiet side	c_3_	0.6253	0.4792	0.7714
	**Effects of >65 dB L_den,most_ combined with a present/absent quiet side on perceived noise control**			
		Coeff.	95%CI lower	95%CI higher
Constant		1.0127	0.5168	1.5086
≤65 dB L_den,most_ + quiet side (ref.)		0		
>65 dB L_den,most_ + quiet side	a_1_	0.5568	0.3901	0.7235
≤65 dB L_den,most_ + no quiet side	a_2_	0.2382	0.1356	0.3408
>65 dB L_den,most_ + no quiet side	a_3_	0.5739	0.3676	0.7801
	**Direct effects of >65 dB L_den,most_ combined with a present/absent quiet side on noise annoyance**			
		Coeff.	95%CI lower	95%CI higher
Constant		0.4493	0.1343	0.7643
≤65 dB L_den,most_ + quiet side (ref.)		0		
>65 dB L_den,most_ + quiet side	c’_1_	0.2064	0.0997	0.3131
≤65 dB L_den,most_ + no quiet side	c’_2_	0.1366	0.0713	0.2018
>65 dB L_den,most_ + no quiet side	c’_3_	0.4418	0.3103	0.5733
Perceived noise control	b	0.3197	0.2905	0.3490
	**Indirect effects through perceived noise control**			
		Coeff.	95%CI lower	95%CI higher
≤65 dB L_den,most_ + quiet side (ref.)		0		
>65 dB L_den,most_ + quiet side	a_1_ × b % ^2^	0.1780 46%	0.1166 30%	0.2554 66%
≤65 dB L_den,most_ + no quiet side	a_2_ × b % ^2^	0.0762 36%	0.0412 29%	0.1164 55%
>65 dB L_den,most_ + no quiet side	a_3_ × b % ^2^	0.1835 29%	0.0980 16%	0.2908 47%

^1^ Based on Model III. ^2^ Percentages given by the formulas (a_1_ × b/c_1_) × 100, (a_2_ × b/c_2_) × 100, (a_3_ × b/c_3_) × 100, respectively.

**Table 9:** Joint associations of exposure to road traffic noise (>65 dB L_den,most_) and present/absent quiet side (intersection in front of living or sleeping room) with perceived noise control and noise annoyance ^1^.

***N*** **= 1812**	**Total Effects of >65 dB L_den,most_ Combined with a Present/Absent Quiet Side on Noise Annoyance**			
		Coeff.	95%CI lower	95%CI higher
Constant		0.7762	0.4273	1.1251
≤65 dB L_den,most_ + quiet side (ref.)		0		
>65 dB L_den,most_ + quiet side	c_1_	0.3143	0.2091	0.4195
≤65 dB L_den,most_ + no quiet side	c_2_	0.5092	0.3422	0.6763
>65 dB L_den,most_ + no quiet side	c_3_	0.7807	0.6041	0.9573
	**Effects of >65 dB L_den,most_ combined with a present/absent quiet side on perceived noise control**			
		Coeff.	95%CI lower	95%CI higher
Constant		1.0421	0.5501	1.5342
≤65 dB L_den,most_ + quiet side (ref.)		0		
>65 dB L_den,most_ + quiet side	a_1_	0.4062	0.2578	0.5546
≤65 dB L_den,most_ + no quiet side	a_2_	0.6721	0.4365	0.9076
>65 dB L_den,most_ + no quiet side	a_3_	0.8504	0.6014	1.0994
	**Direct effects of >65 dB L_den,most_ combined with a present/absent quiet side on noise annoyance**			
		Coeff.	95%CI lower	95%CI higher
Constant		0.4479	0.1337	0.7621
≤65 dB L_den,most_ + quiet side (ref.)		0		
>65 dB L_den,most_ + quiet side	c’_1_	0.1863	0.0913	0.2814
≤65 dB L_den,most_ + no quiet side	c’_2_	0.2975	0.1465	0.4485
>65 dB L_den,most_ + no quiet side	c’_3_	0.5128	0.3526	0.6731
Perceived noise control	b	0.3150	0.2856	0.3444
	**Indirect effects through perceived noise control**			
		Coeff.	95%CI lower	95%CI higher
≤65 dB L_den,most_ + quiet side (ref.)		0		
>65 dB L_den,most_ + quiet side	a_1_ × b ^2^ %	0.1280 41%	0.0744 24%	0.1955 62%
≤65 dB L_den,most_ + no quiet side	a_2_ × b ^2^ %	0.2117 42%	0.1050 21%	0.3491 69%
>65 dB L_den,most_ + no quiet side	a_3_ × b ^2^ %	0.2679 34%	0.1626 21%	0.4016 51%

^1^ based on Model III. ^2^ Percentages given by the formulas (a_1_ × b/c_1_) × 100, (a_2_ × b/c_2_) × 100, (a_3_ × b/c_3_) × 100, respectively.

**Table 10:** Joint associations of exposure to road traffic noise (>65 dB L_den,most_) and present/absent quiet side (>40 dB L_den,least_) with perceived noise control and noise annoyance ^1^.

***N*** **= 1812**	**Total Effects of >65 dB L_den,most_ Combined with a Present/Absent Quiet Side on Noise Annoyance**			
		Coeff.	95%CI lower	95%CI higher
Constant		0.7428	0.3900	1.0956
≤65 dB L_den,most_ + quiet side (ref.)		0		
>65 dB L_den,most_ + quiet side	c_1_	0.2948	0.1697	0.4199
≤65 dB L_den,most_ + no quiet side	c_2_	0.1423	0.0573	0.2272
>65 dB L_den,most_ + no quiet side	c_3_	0.5877	0.4546	0.7207
	**Effects of >65 dB L_den,most_ combined with a present/absent quiet side on perceived noise control**			
		Coeff.	95%CI lower	95%CI higher
Constant		1.0204	0.5230	1.5178
≤65 dB L_den,most_ + quiet side (ref.)		0		
>65 dB L_den,most_ + quiet side	a_1_	0.4077	0.2313	0.5842
≤65 dB L_den,most_ + no quiet side	a_2_	0.1217	0.0019	0.2415
>65 dB L_den,most_ + no quiet side	a_3_	0.6160	0.4284	0.8036
	**Direct effects of >65 dB L_den,most_ combined with a present/absent quiet side on noise annoyance**			
		Coeff.	95%CI lower	95%CI higher
Constant		0.4133	0.0977	0.7289
≤65 dB L_den,most_ + quiet side (ref.)		0		
>65 dB L_den,most_ + quiet side	c’_1_	0.1632	0.2937	0.3521
≤65 dB L_den,most_ + no quiet side	c’_2_	0.1030	0.0511	0.2752
>65 dB L_den,most_ + no quiet side	c’_3_	0.3888	0.2689	0.5086
Perceived noise control	b	0.3229	0.2937	0.3521
	**Indirect effects through perceived noise control**			
		Coeff.	95%CI lower	95%CI higher
≤65 dB L_den,most_ + quiet side (ref.)		0		
>65 dB L_den,most_ + quiet side	a_1_ × b % ^2^	0.1317 45%	0.0687 23%	0.2097 71%
≤65 dB L_den,most_ + no quiet side	a_2_ × b % ^2^	0.0393 32%	−0.0001 0%	0.0842 69%
>65 dB L_den,most_ + no quiet side	a_3_ × b % ^2^	0.1989 34%	0.1211 21%	0.2943 50%

^1^ Based on Model III. ^2^ Percentages given by the formulas (a_1_ × b/c_1_) × 100, (a_2_ × b/c_2_) × 100, (a_3_ × b/c_3_) × 100, respectively.

**Table 11:** Joint associations of exposure to road traffic noise (>65 dB L_den,most_) and present/absent dwelling-related green with perceived noise control and noise annoyance ^1^.

***N*** **= 1786**	**Total Effects of >65 dB L_den,most_ Combined with Present/Absent Dwelling-Related Green on Noise Annoyance**			
		Coeff.	95%CI lower	95%CI higher
Constant		0.7115	0.3538	1.0692
≤65 dB L_den,most_ + green (ref.)		0		
>65 dB L_den,most_ + green	c_1_	0.3698	0.2564	0.4832
≤65 dB L_den,most_ + no green	c_2_	0.1554	0.0696	0.2413
>65 dB L_den,most_ + no green	c_3_	0.5952	0.4320	0.7584
	**Effects of >65 dB L_den,most_ combined with present/absent dwelling-related green on perceived noise control**			
		Coeff.	95%CI lower	95%CI higher
Constant		0.9814	0.4783	1.4845
≤65 dB L_den,most_ + green (ref.)		0		
>65 dB L_den,most_ + green	a_1_	0.3930	0.2335	0.5525
≤65 dB L_den,most_ + no green	a_2_	0.1341	0.0133	0.2548
>65 dB L_den,most_ + no green	a_3_	0.8022	0.5727	1.0318
	**Direct effects of >65 dB L_den,most_ combined with present/absent dwelling-related green on noise annoyance**			
		Coeff.	95%CI lower	95%CI higher
Constant		0.3953	0.0750	0.7155
≤65 dB L_den,most_ + green (ref.)		0		
>65 dB L_den,most_ + green	c’_1_	0.2432	0.1415	0.3450
≤65 dB L_den,most_ + no green	c’_2_	0.1122	0.0356	0.1889
>65 dB L_den,most_ + no green	c’_3_	0.3368	0.1893	0.4842
Perceived noise control	b	0.3222	0.2926	0.3517
	**Indirect effects through perceived noise control**			
		Coeff.	95%CI lower	95%CI higher
≤65 dB L_den,most_ + green (ref.)		0		
>65 dB L_den,most_ + green	a_1_ × b % ^2^	0.1266 34%	0.0772 21%	0.2099 57%
≤65 dB L_den,most_ + no green	a_2_ × b % ^2^	0.0432 28%	0.0054 4%	0.0859 55%
>65 dB L_den,most_ + no green	a_3_ × b % ^2^	0.2585 43%	0.1528 27%	0.3867 65%

^1^ Based on Model III. ^2^ Percentages given by the formulas (a_1_ × b/c_1_) × 100, (a_2_ × b/c_2_) × 100, (a_3_ × b/c_3_) × 100, respectively.
